# Gerard M. Guiraudon: Paris 1931—London, Ontario 2019

**DOI:** 10.1007/s12471-019-01329-5

**Published:** 2019-09-17

**Authors:** N. van Hemel, F. Vemeulen, J. Defauw, J. de Bakker, H. Kingma, J. Ernst

**Affiliations:** 1 Odijk, The Netherlands; 2 Wilrijk, Belgium; 3 Wijk bij Duurstede, The Netherlands; 4 Muiden, The Netherlands; 5 Delden, The Netherlands; 6 Bilthoven, The Netherlands


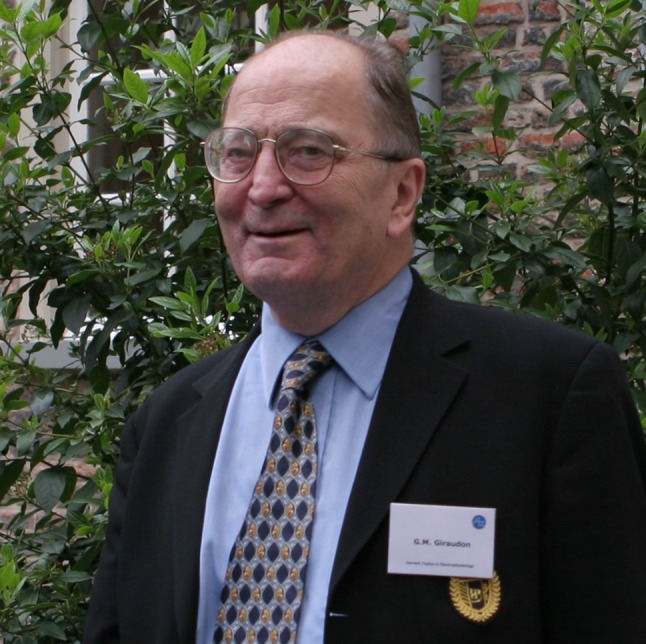


Recently we received the sad news that Gerard Guiraudon, one of the pioneers of cardiac arrhythmic surgery in the second half of the 20th century, passed away in February 2019. Guiraudon received his medical training in Paris and became Professor of Anatomy at Sorbonne University. He received his surgical training and was a member of the team performing the first heart transplant in Europe at the famous Salpêtrière Hospital in Paris in April 1968.

Guiraudon specifically explored the role of cardiac surgery in the treatment of all types of cardiac arrhythmias. For ischaemic ventricular tachycardia he designed, in collaboration with Guy Fontaine, the endocardial encircling ventriculotomy, excluding zones of the tachycardia to terminate ventricular tachycardia. His concept of corridor surgery for atrial fibrillation included the transmission of the sinus node impulse through the right atrium to the atrioventricular node, whereas the left atrium was allowed to harbour ongoing atrial fibrillation. Regarding the Wolff-Parkinson-White syndrome Guiraudon was able to dissect and cut the accessory fibres on the beating heart. His ultimate surgical goal was to carry out arrhythmia ‘surgery without surgery’, meaning minimally invasive procedures. His thoughts on mechanisms and radical treatment of arrhythmia have been translated in the past into many catheter-mediated approaches with definitive arrhythmic suppression and maximal patient comfort.

From 1970 to 2003 Guiraudon, during that time affiliated with the London Ontario University (Canada), frequently visited the St. Antonius Hospital Nieuwegein/Utrecht to perform complex arrhythmia surgery, thereby teaching how to handle medically untreatable cases. We celebrated successes together but at the same time had to accept inevitable failures. This experience and innovative surgical concepts spread to other Dutch surgical centres. To express our gratitude for his efforts on behalf of Dutch patients and our profession, in February 2005 the symposium ‘Exclusion or Targeting’ was organised in Utrecht which encompassed the opinions, experience and scientific results of many national experts. On that occasion he received a high royal distinction.

Gerard Guiraudon had an extraordinary personality. He had an excellent anatomical knowledge, based on lifelong discussions with his wife Prof. Colette Guiraudon, who was an excellent pathologist and chaired the Pathology Department of the University of Western Ontario, London, Canada. Moreover, he could talk endlessly about history, philosophy and literature, based on a wealth of knowledge collected during his youth in France and the years thereafter.

Talking with Gerard was exhausting not only because of his overwhelming views and insights but also because of the endurance of his points of view. Apart from his enormous cognitive capacity and fluency of speech he was deeply saddened by medical failures, a reason to love this surgeon who had a considerable feeling of responsibility toward himself and his coworkers. Gerard Guiraudon will be remembered as a personality with a warm heart, brilliant brain and for his numerous innovative concepts. We send our warmest condolences to his three children, family and friends.

